# High expression of *TMEM244* is associated with poor overall survival of patients with T-cell lymphoma

**DOI:** 10.1186/s40364-022-00395-z

**Published:** 2022-07-12

**Authors:** Cunte Chen, Shaohua Chen, Gengxin Luo, Liang Wang, Chengwu Zeng, Grzegorz K. Przybylski, Yangqiu Li

**Affiliations:** 1 Institute of Hematology, School of Medicine, Key Laboratory for Regenerative Medicine of Ministry of Education, Jinan University, Guangzhou, China; 2 Department of Hematology, First Affiliated Hospital, Jinan University, Guangzhou, China; 3 Department of Oncology, First Affiliated Hospital, Jinan University, Guangzhou, China; 4 Institute of Human Genetics, Polish Academy of Sciences, Poznań, Poland

**Keywords:** TMEM244, Prognosis, T-cell lymphoma, T-cell acute lymphoblastic leukemia

## Abstract

T-cell lymphoma (TCL) is an aggressive and genetically heterogeneous malignancy with adverse clinical outcomes; thus, it is worth exploring biomarkers for risk stratification. Previous studies have demonstrated that transmembrane protein 244 gene (*TMEM244*) is ectopically expressed in Sézary syndrome (SS). In this study, the expression level of *TMEM244* and its prognostic value for TCL patients was explored by analyzing RNA-seq data of two large datasets (GSE132550 and GSE113113) containing 129 TCL patients and 13 healthy individuals (HIs) from the Gene Expression Omnibus (GEO) database, the PRJCA002270 dataset containing 124 patients with T-cell acute lymphoblastic leukemia (T-ALL) from the BioProject database, and peripheral blood (PB) samples of 24 TCL and 29 T-ALL patients, as well as 11 normal CD3 + T-cells from our clinical center (JNU). The results suggested that *TMEM244* was significantly up-regulated in TCL patients compared with normal CD3 + T-cells or T-ALL in the JNU, GSE132550 and GSE113113 datasets (*P* < 0.05). However, *TMEM244* shows no expression in patients with T-ALL in the JNU-T-ALL and PRJCA002270 datasets. The receiver operating characteristic (ROC) curve analysis indicated that *TMEM244* expression had a very high accuracy in diagnosing TCL compared with T-ALL (area under the curve (AUC): 99.4%; *P* < 0.001). Importantly, high *TMEM244* expression was significantly associated with poor OS and shorter 5-year restricted mean survival time (RMST) in TCL patients, especially those treated with chemotherapy. In summary, *TMEM244* is also expressed in other types of TCL besides SS, but not in T-ALL. High *TMEM244* expression is associated with poor OS in TCL patients, which might be a novel biomarker for prognostic stratification in TCL patients and facilitate the design of novel therapies.

**Supplementary Information:**

The online version contains supplementary material available at 10.1186/s40364-022-00395-z.

**To the Editor**,

T-cell lymphoma (TCL) originates from lymphoblastoid or mature T cells and accounts for 10–15% of non-Hodgkin lymphoma, which can be further subdivided into various subtypes [1, 2]. TCL is a highly aggressive malignancy that exhibits hematologic and prognostic heterogeneity [3]. In contrast to the tremendous progress in the treatment of B-cell lymphoma, the treatment of TCL is still not very effective in most cases with a 5-year overall survival (OS) of less than 50% [4, 5]. However, current risk stratification based on the international prognostic index (including age, Ann Arbor stage, performance status, serum lactate dehydrogenase level, and extranodal involvement) cannot accurately predict the prognosis of all TCL patients [6, 7]. However, genetics in TCL are lacking for supplementing risk stratification to relatively accurately predict the prognosis of TCL patients [6, 8–10]. In our previous publications, we found that transmembrane protein 244 gene (*TMEM244*) is ectopically expressed in Sézary syndrome (SS), driven by hypomethylation of the promoter region [11, 12]. However, the biological function, and prognostic and diagnostic significance of *TMEM244* expression in TCL patients remain unknown.

In this study, transcriptome sequencing data of two large datasets (GSE132550 and GSE113113) containing 129 TCL patients and 13 healthy individuals (HIs) from the Gene Expression Omnibus (GEO) database, and the PRJCA002270 dataset containing 124 patients with T-cell acute lymphoblastic leukemia (T-ALL) from the BioProject database were downloaded. Peripheral blood (PB) samples of 24 TCL and 29 T-ALL patients, as well as 11 normal CD3 + T-cells from our Jinan University (JNU) clinical center were used to investigate the expression level of *TMEM244* and its prognostic value for TCL patients (Table S1). *TMEM244* expression in PB samples from JNU was detected by quantitative real-time PCR (qRT-PCR). We first found that *TMEM244* was significantly up-regulated in TCL patients compared with normal CD3 + T-cells or T-ALL in the JNU dataset (*P* < 0.001, Fig. 1A). Similar results were obtained in the GSE132550 and GSE113113 datasets (*P* < 0.05, Fig. 1A). However, compared with normal CD3 + T-cells, *TMEM244* shows low trace expression in 4 out of 29 (13.8%) patients with T-ALL, while no expression was detected in the remained 25 (86.2%) T-ALL patients in the JNU-T-ALL dataset (*P* < 0.001; Fig. 1A-B). These results were confirmed in the PRJCA002270 dataset (trace vs. no *TMEM244* expression: 5/124 (4.0%) vs. 119/124 (96.0%)) (Fig. 1B). To evaluate the sensitivity and accuracy of high *TMEM244* expression in diagnosing TCL, we performed a receiver operating characteristic (ROC) curve analysis in the JNU dataset. The results indicated that *TMEM244* expression had a very high accuracy in diagnosing TCL compared with T-ALL (area under the curve (AUC): 99.4%, 95% confidence interval (CI): 98.2-100%; *P* < 0.001) (Fig. 1C). We further obtained the optimal cut-point 1.5 in the ROC, suggesting that its sensitivity in diagnosing TCL was as high as 95.8% (Fig. 1C), when the expression level of *TMEM244* was greater than 1.5. Moreover, when the expression level of TMEM244 was lower than 1.5, 100% of T-ALL was diagnosed correctly, while 4.2% mis-diagnosis was observed in TCL (Fig. 1D). These results suggested that *TMEM244* showed high expression in TCL derived from mature T-cells and virtually no expression in T-ALL derived from immature T cells. The trace expression in 4/29 (JNU) and 5/124 (PRJCA002270) T-ALL might be from the remaining normal T cells.

**Fig. 1:**
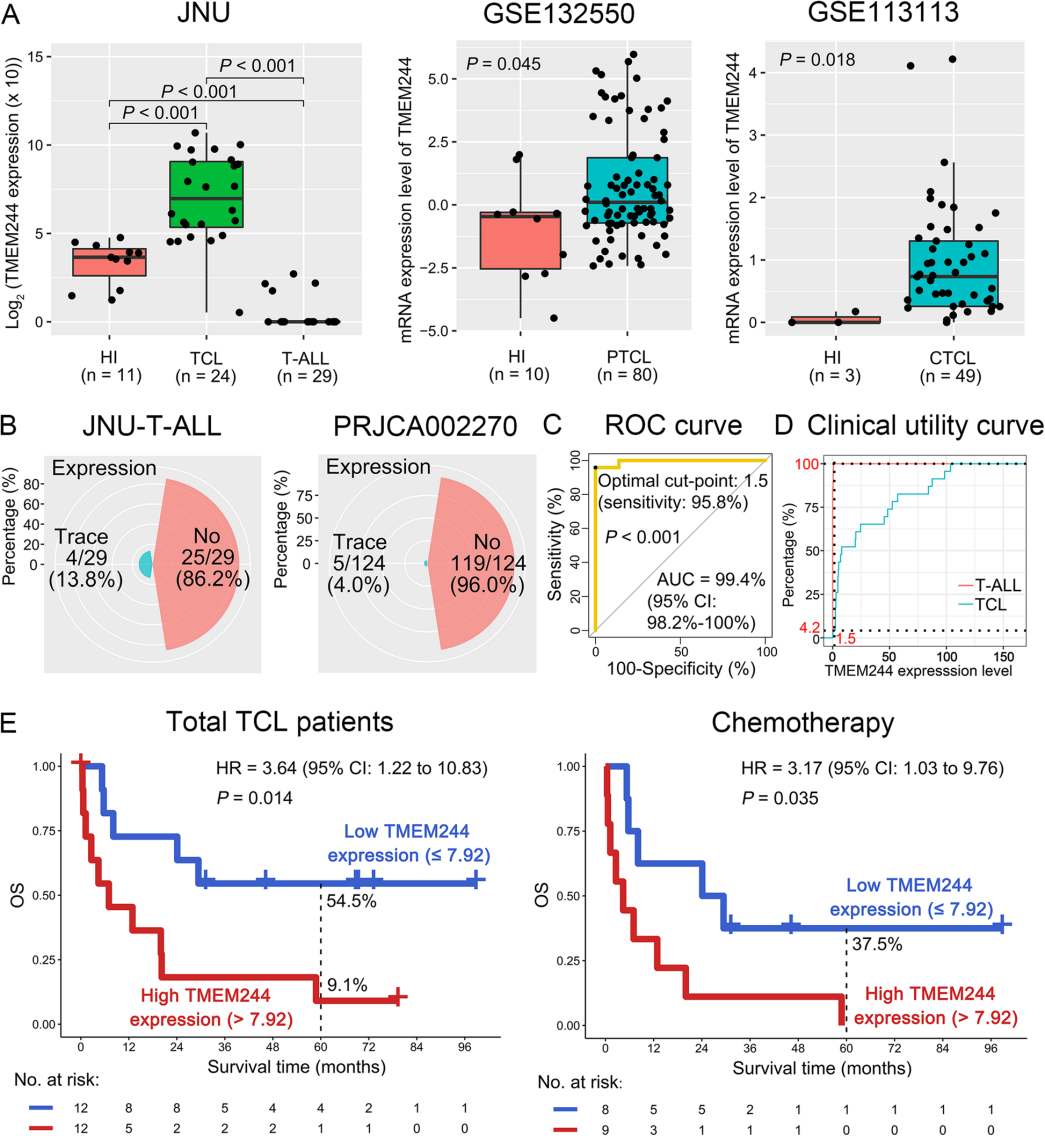
The expression level of TMEM244 in TCL and T-ALL, and its prognostic value in TCL patients. **A** TMEM244 expression levels in healthy individuals (HIs) and patients with TCL or T-ALL in the JNU (left panel), GSE132550 (middle panel), and GSE113113 (right panel) datasets. **B** Percentage in T-ALL patients with no or trace expression of TMEM244 in the JNU-T-ALL (left panel) and PRJCA002270 (right panel) datasets. **C** The sensitivity and accuracy of high TMEM244 expression in diagnosing TCL compared with T-ALL. **D** Clinical utility curve for diagnosing TCL and T-ALL in the JNU dataset. **E** The overall survival (OS) for the low and high TMEM244 expression subgroups in the total TCL patients (left panel) and patients treated with chemotherapy (right panel) in the JNU-TCL dataset. AUC: Area under the curve; CI: Confidence interval; CTCL: Cutaneous T-cell lymphoma; HR: Hazard ratio; PTCL: Peripheral T-cell lymphoma; ROC: Receiver operating characteristic

To elucidate the prognostic importance of the *TMEM244* expression in TCL patients, Kaplan-Meier survival analysis was performed. According to an optimal cut-point of 7.92, TCL patients in the JNU dataset were divided into two subgroups: low and high *TMEM244* expression (Fig. S1). High *TMEM244* expression was significantly associated with poor OS in patients with TCL (hazard ratio (HR) = 3.64, 95% confidence interval (CI): 1.22–10.83; 5-year OS: 9.1% vs. 54.5%; *P* = 0.014) (Fig. 1E). Further subgroup analysis indicated that high *TMEM244* expression predicted poor OS in TCL patients who were treated with chemotherapy only (HR = 3.17, 95% CI: 1.03–9.76; 5-year OS: 0% vs. 37.5%; *P* = 0.035) (Fig. 1E). However, the expression level of *TMEM244* was not significantly correlated with OS for TCL patients who underwent hematopoietic stem cell transplantation (HSCT), suggesting that HSCT could overcome this poor genetic alteration (*P* = 0.221, Fig. S2). Furthermore, patients with high *TMEM244* expression had a shorter restricted mean survival time (RMST) than those with low *TMEM244* expression (5-year RMST: 39.3 (95% CI: 25.3–53.3) vs. 17.1 (95% CI: 4.6–29.6) months; Fig. S3A). Similar results were shown in patients with chemotherapy only, but not HSCT (5-year RMST: 31.6 (95% CI: 15.3–47.8) vs. 12.0 (95% CI: 0.5–23.5) months; Fig. S3B-C). However, the small number size in TCL patients treated with HSCT might lead to statistical biases, further investigation with large cohort is needed to confirm the finding. Importantly, when gender, age, treatment options, and *TMEM244* expression were included in the univariate and multivariate Cox regression models analysis, the results indicated that high *TMEM244* expression was an independent prognostic predictor for OS of TCL patients (HR = 5.43; 95% CI: 1.64–18.01; *P* = 0.006) (Table 1). These results demonstrate that up-regulation of *TMEM244* might play a critical role in the prognosis of TCL patients. Nevertheless, more TCL cohorts are needed for validation of the findings in the future.

**Table 1:** Univariate and multivariate Cox regression analysis in TCL patients

Variables^a^	Univariate Cox		Multivariate Cox	
	HR (95% CI)	*P* value	HR (95% CI)	*P* value
TMEM244				
Low expression	Reference		Reference	
High expression	3.64 (1.22–10.83)	**0.020**	5.43 (1.64–18.01)	**0.006**
Gender				
Female	Reference		Reference	
Male	0.43 (0.15–1.20)	0.107	0.42 (0.13–1.40)	0.158
Age, years	1.02 (0.99–1.05)	0.198	1.02 (0.98–1.06)	0.335
Treatment				
Chemotherapy	Reference		Reference	
HSCT	0.12 (0.02–0.93)	0.042	0.25 (0.02–2.59)	0.245

*CI* Confidence interval, *HR* Hazard ratio, *HSCT* Hematopoietic stem cell transplantation

^a^Analysis of TCL patients with complete clinical information

In conclusion, we reveal that besides SS, *TMEM244* is also expressed in other types of TCL, but not in T-ALL. High *TMEM244* expression is significantly associated with poor OS in TCL patients, which might be a novel biomarker for prognostic stratification in TCL patients.

## Supplementary Information

**Additional file 1.** Materials and methods. **Fig. S1.** The optimal cut-point for TMEM244 expression in TCL patients in the JNU-TCL dataset. **Fig. S2.** OS analysis of TMEM244 in TCL patients treated with hematopoietic stem cell transplantation (HSCT) in the JNU-TCL dataset. **Fig. S3.** The 5-year restricted mean survival time (RMST) for the low and high TMEM244 expression subgroups in the total TCL patients (upper panel), patients treated with chemotherapy (middle panel), or HSCT (bottom panel) in the JNU-TCL dataset. **Table S1.** Clinical information of patients with TCL and T-ALL.

## Data Availability

The GSE132550 and GSE113113 datasets were downloaded from the Gene Expression Omnibus (GEO) dataset (https://www.ncbi.nlm.nih.gov/geo/). The PRJCA002270 dataset used in this study were acquired from the BioProject database (https://ngdc.cncb.ac.cn/bioproject/browse/PRJCA002270). The datasets used and/or analyzed during the current study are available from the corresponding author on reasonable request.

## Abbreviations

AUC: Area under the curve
CI: Confidence interval
GEO: Gene Expression Omnibus
HR: Hazard ratio
HSCT: Hematopoietic stem cell transplantation
JNU: Jinan University
OS: Overall survival
PB: Peripheral blood
qRT-PCR: Quantitative real-time PCR
RMST: Restricted mean survival time
ROC: Receiver operating characteristic
SS: Sézary syndrome
T-ALL: T-cell acute lymphoblastic leukemia
TCL: T-cell lymphoma
TMEM244: Transmembrane protein 244 gene
