# Supplementary material for: High expression of TMEM244 is associated with poor overall survival of patients with T-cell lymphoma
Source: Biomark Res. 2022 Jul 12;10:46. doi: 10.1186/s40364-022-00395-z (PMC9281042; doi:10.1186/s40364-022-00395-z)
Supplement: Supplementary file 1 — Additional file 1. Materials and methods. Fig. S1. The optimal cut-point for TMEM244 expression in TCL patients in the JNU-TCL dataset. Fig. S2. OS analysis of TMEM244 in TCL patients treated with hematopoietic stem cell transplantation (HSCT) in the JNU-TCL dataset. Fig. S3. The 5-year restricted mean survival time (RMST) for the low and high TMEM244 expression subgroups in the total TCL patients (upper panel), patients treated with chemotherapy (middle panel), or HSCT (bottom panel) in the JNU-TCL dataset. Table S1. Clinical information of patients with TCL and T-ALL. [file 40364_2022_395_MOESM1_ESM.doc]

**High expression of *TMEM244* is associated with poor overall survival of patients with T-cell lymphoma**

Cunte Chen^1^, Shaohua Chen^1^, Gengxin Luo^2^, Liang Wang^3^, Chengwu Zeng^1 *^, Grzegorz K. Przybylski^4 *^, Yangqiu Li^1 *^

^1^ Institute of Hematology, School of Medicine, Key Laboratory for Regenerative Medicine of Ministry of Education, Jinan University, Guangzhou, China.

^2^ Department of Hematology, First Affiliated Hospital, Jinan University, Guangzhou, China.

^3^ Department of Oncology, First Affiliated Hospital, Jinan University, Guangzhou, China.

^4^ Institute of Human Genetics, Polish Academy of Sciences, Poznań Poland.

***** Corresponding author:

E-mail addresses:

Yangqiu Li: [yangqiuli@hotmail.com](mailto:yangqiuli@hotmail.com)

Grzegorz K. Przybylski: [grzegorz.przybylski@igcz.poznan.pl](mailto:grzegorz.przybylski@igcz.poznan.pl)

Chengwu Zeng: [bio-zcw@163.com](mailto:bio-zcw@163.com)

**Materials and methods**

**TCL and T-ALL patients**

Peripheral blood (PB) samples from 24 TCL and 29 T-ALL patients were collected at Jinan University (JNU) between December 30, 2009, and March 1, 2022. Corresponding clinical information, including gender, age, histopathological subtypes, treatment options, OS time, and event, were also collected (Table S1). The last follow-up date for TCL patients was April 1, 2022, and the median follow-up time for surviving TCL patients was 69.4 months (range: 31.2-98.8 months). A consent form was obtained from each participant following the guidelines of the Institutional Review Board requirements and Declaration of Helsinki principles. This study was approved by the Ethical Committee of Jinan University.

Transcriptome sequencing data of 80 Formalin-Fixed and Paraffin-Embedded (FFPE) samples from peripheral TCL (PTCL) and 10 healthy individuals (HIs) from the GSE132550 dataset [1] and 49 skin situ tissues samples from cutaneous T-cell lymphoma (CTCL) and 3 HIs from the GSE113113 dataset [2, 3] were downloaded from the Gene Expression Omnibus (GEO) dataset (<https://www.ncbi.nlm.nih.gov/geo/>). Moreover, transcriptome sequencing data of 124 T-ALL patients in the PRJCA002270 dataset, were obtained from the BioProject database (<https://ngdc.cncb.ac.cn/bioproject/browse/> PRJCA002270) [4]. The GSE132550, GSE113113, and PRJCA002270 datasets are publicly available; thus, approval from a local ethics committee was not required.

**CD3+ T-cells were selected from PBMCs of HIs**

CD3+ T-cells were positively selected from PB mononuclear cells (PBMCs) of 11 HIs using human CD3 microbeads (Miltenyi Biotec, Bergisch. Gladbach, Germany) according to the manufacturer's instructions [5].

**Quantitative real-time polymerase chain reaction (qRT-PCR)**

qRT-PCR was performed using the CFX Connect Real-Time System (Bio-Rad, Hercules, CA, USA). *TMEM244* expression in HIs, TCL, and T-ALL patients was analyzed using TaqMan Gene Expression Assays (Applied Biosystems, Foster City, CA, USA) (Hs02340633_m1) [6]. GAPDH was used as an internal control, which was detected by TaqMan Gene Expression Assays (Applied Biosystems, Foster City, CA, USA) (Hs03929097_g1). qRT-PCR reaction procedures were set as follows: Initial activation, 95 °C for 15 min; and amplification, 95 °C for 15 sec and 60 °C for 1 min, for a total of 40 cycles. The results are presented as 2^−ΔΔCT^.

**Statistical analysis**

Statistical analyses were performed using SPSS 22.0 (IBM, Armonk, New York, USA) and R (version 4.0.2, <https://www.r-project.org/>), as appropriate. The optimal cut-points for TMEM244 expression were obtained by the R package “survminer” [5, 7, 8]. Differences in subgroups in Kaplan-Meier curves were compared using the log-rank test in the R package "survival" [9, 10]. The restricted mean survival time (RMST) was determined by R package "survRM2" [11, 12]. Differences in 2 independent groups were tested by Wilcoxon. The area under the curve (AUC) in the receiver operating characteristic (ROC) curve was determined by the R package "pROC" [13]. Clinical utility curve was plotted using the R packages "ggpubr", " data.table", "pROC", and "eoffice". Univariate and multivariate Cox regression analyses were performed using SPSS software. Two-tailed *P* < 0.05 was considered statistically significant.

**References**


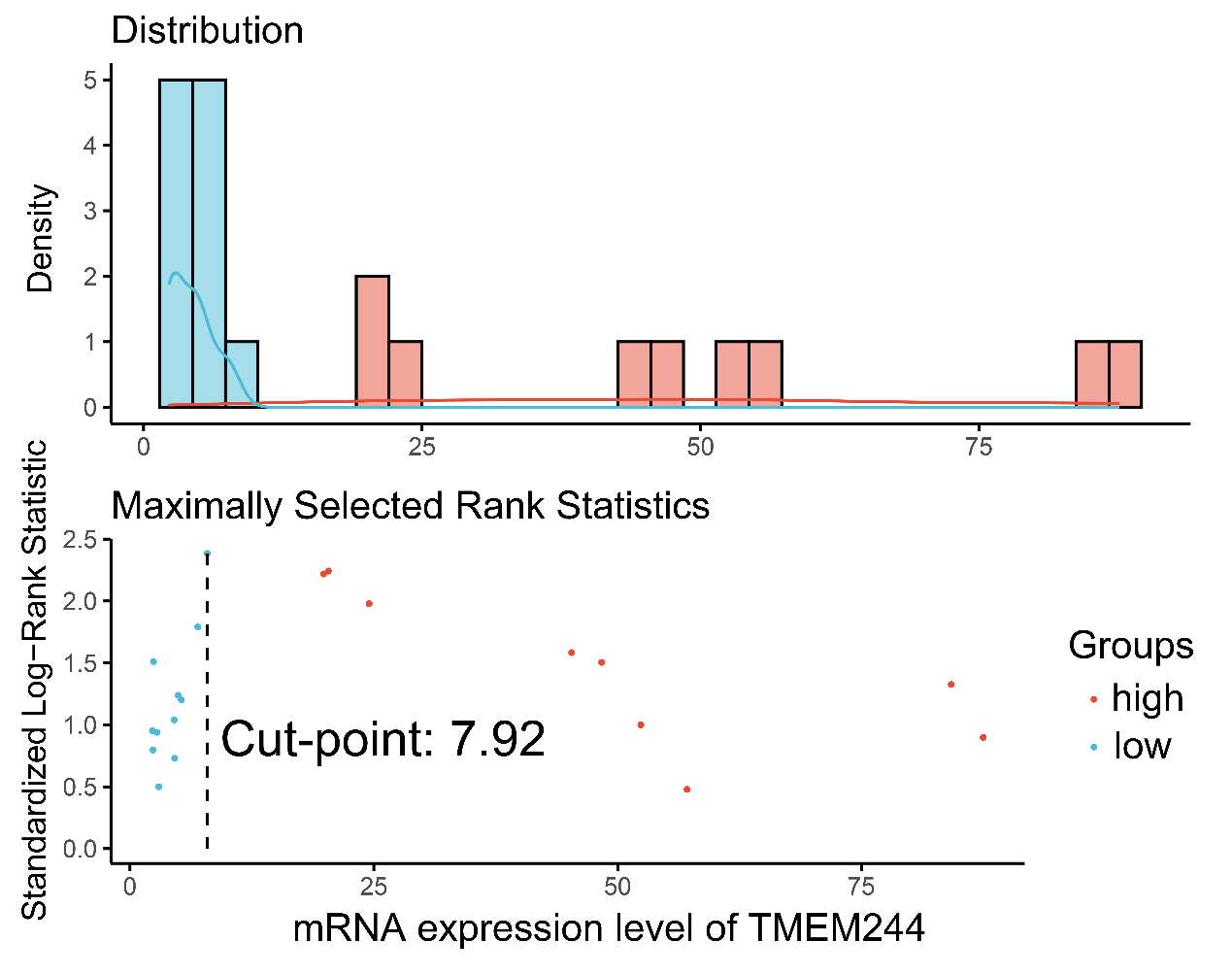


**Fig. S1** The optimal cut-point for *TMEM244* expression in TCL patients in the JNU-TCL dataset.


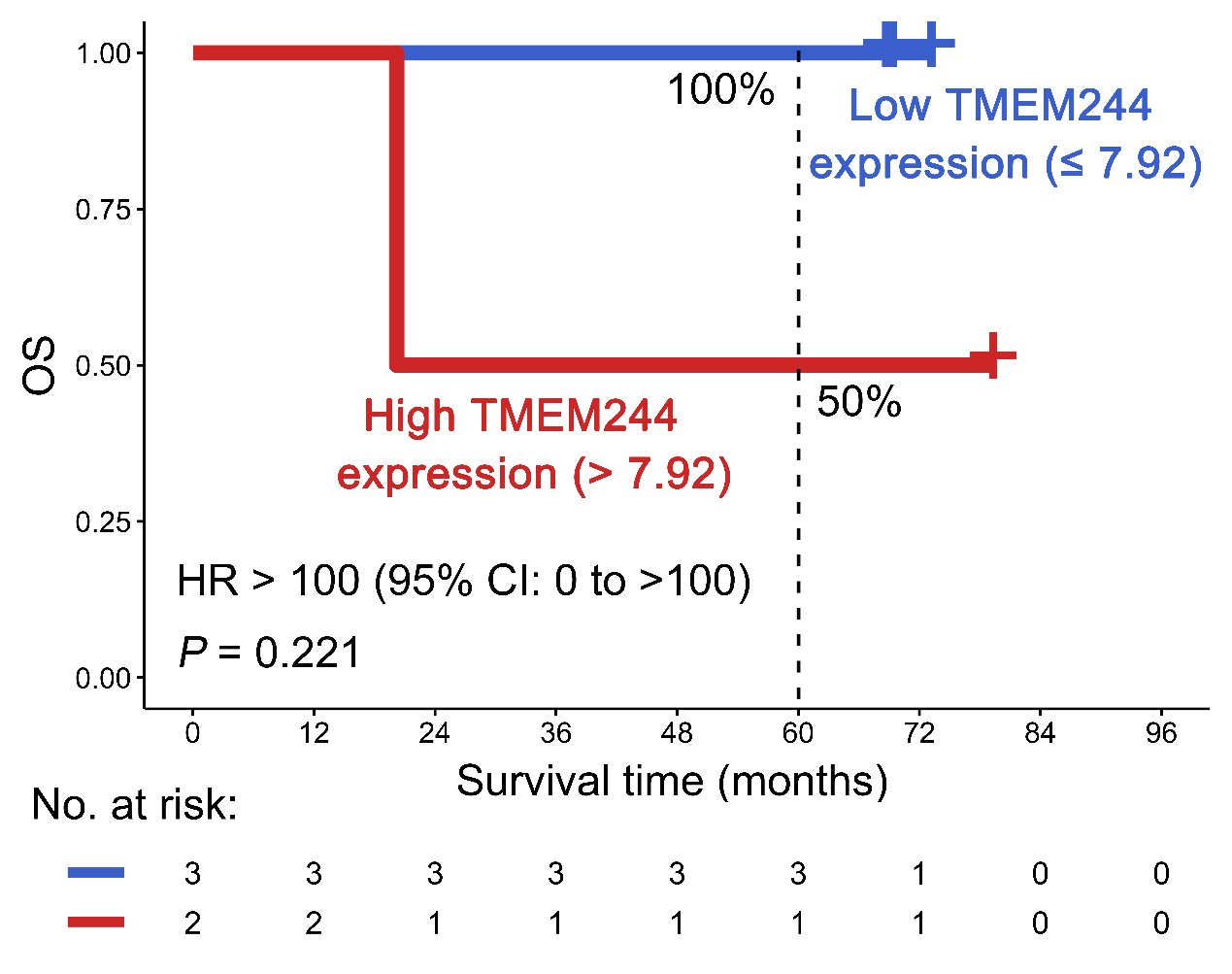


**Fig. S2** OS analysis of *TMEM244* in TCL patients treated with hematopoietic stem cell transplantation (HSCT) in the JNU-TCL dataset.


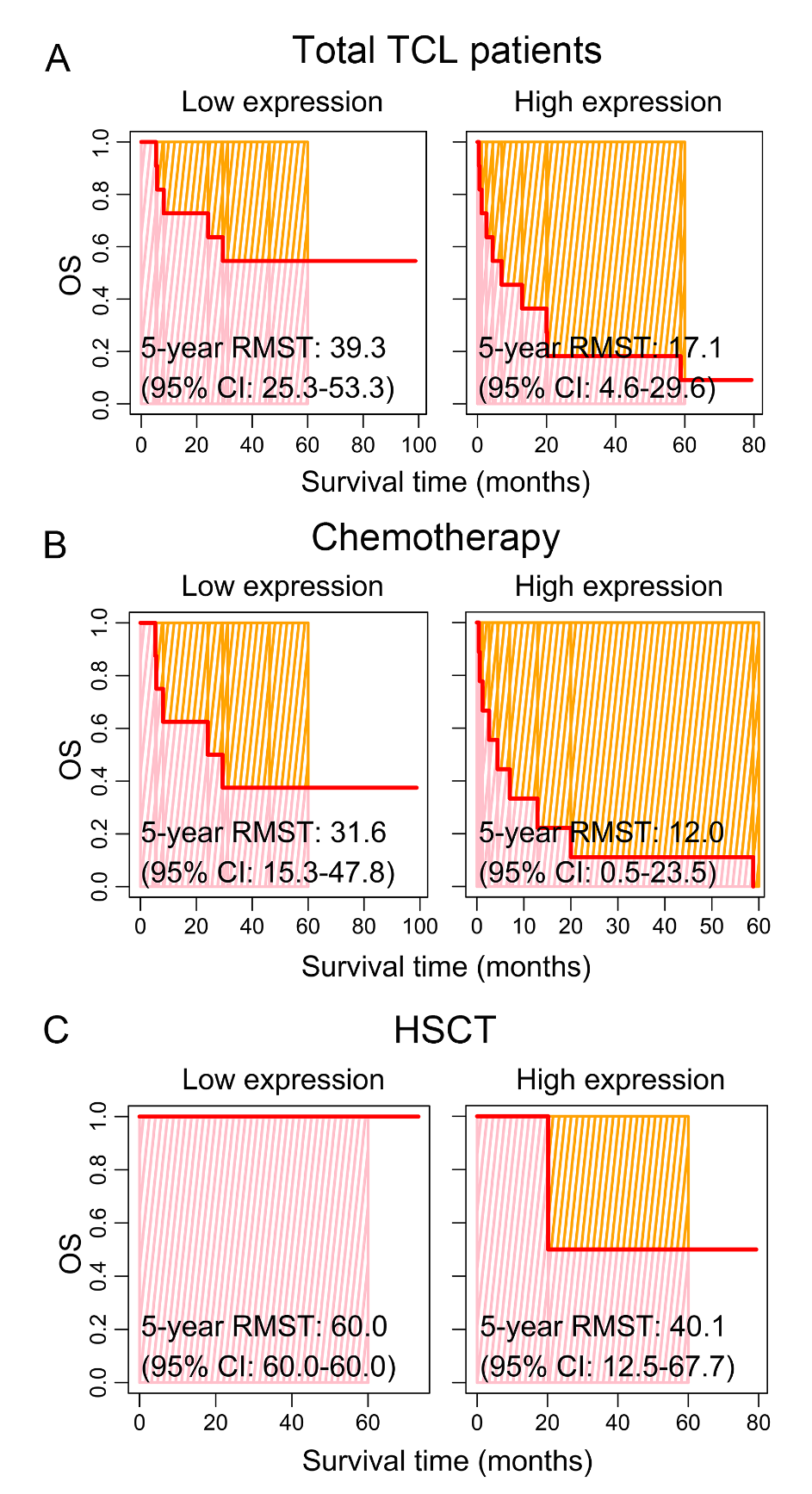


**Fig. S3** The 5-year restricted mean survival time (RMST) for the low and high *TMEM244* expression subgroups in the total TCL patients (upper panel), patients treated with chemotherapy (middle panel), or HSCT (bottom panel) in the JNU-TCL dataset.

**Table S1.** Clinical information of patients with TCL and T-ALL.

| Variable | JNU-TCL | JNU-T-ALL | GSE132550 | GSE113113 | PRJCA002270 |
| --- | --- | --- | --- | --- | --- |
| Number | 24 | 29 | 80 | 49 | 124 |
| Age, years,  mean ± SD | 39.4 ± 18.6 | 30 ± 13.9 | - | - | 15.2 ± 13.8 |
| Sample source | PB | PB | FFPE tumor sample | Skin tissue | - |
| Gender (%) |  |  |  |  |  |
| Female | 12 (50.0) | 9 (31.0) | - | - | 33 (26.6) |
| Male | 12 (50.0) | 20 (69.0) | - | - | 90 (72.6) |
| Unkown | 0 (0) | 0 (0) | - | - | 1 (0.8) |
| Subtype (%) * |  |  |  |  |  |
| T-ALL | - | 29 (100) | - | - | 124 (100) |
| AITL | 2 (8.3) | - | - | - | - |
| ALCL | 1 (4.2) | - | - | - | - |
| CTCL | 6 (25.0) | - | - | 49 (100) | - |
| EATL | 1 (4.2) | - | - | - | - |
| GDTCL | 1 (4.2) | - | - | - | - |
| HSTCL | 1 (4.2) | - | - | - | - |
| NKTCL | 2 (8.3) | - | - | - | - |
| PTCL-NOS | 5 (20.8) | - | 68 (85.0) | - | - |
| T-LBL | 5 (20.8) | - | - | - | - |
| LL | 0 (0) | - | 12 (15.0) | - | - |
| Treatment (%) * |  |  |  |  |  |
| Chemotherapy | 17 (70.8) | - | - | - | - |
| HSCT | 5 (20.8) | - | - | - | - |
| Other | 2 (8.3) | - | - | - | - |

* Due to rounding, not all percentages total 100%

AITL: Angioimmunoblastic T cell lymphoma; ALCL: Anaplastic large cell lymphoma; CTCL: Cutaneous T-cell lymphoma; EATL: Enteropathy-associated T-Cell Lymphoma; FFPE: Formalin-Fixed, Paraffin-Embedded; GDTCL: γδ-T-cell lymphoma; HSCT: Hematopoietic stem cell transplantation; HSTCL: Hepatosplenic T cell lymphoma; LL: Lennert Lymphoma; NKTCL: NK-T cell lymphoma; PB: Peripheral blood; PTCL-NOS: Peripheral T cell lymphoma, not otherwise specified (NOS); SD: Standard deviation; T-LBL: T-lymphoblastic lymphoma.
